# Supplementary material for: Digital Online Anaesthesia Patient Informed Consent before Elective Diagnostic Procedures or Surgery: Recent Practice in Children—An Exploratory ESAIC Survey (2021)
Source: J Clin Med. 2022 Jan 19;11(3):502. doi: 10.3390/jcm11030502 (PMC8836584; doi:10.3390/jcm11030502)
Supplement: Supplementary file 1 [file jcm-11-00502-s001.zip › jcm-1510923-supplementary.pdf]

# Supplementary Material

**Table S1.** Number of responses.

| Country of Employment? n (%) | | | |
|---|---|---|---|
| Albania | 3 (0.3) | Liechtenstein | 1 (0.1) |
| Austria | 32 (3.4) | Lithuania | 8 (0.9) |
| Belarus | 2 (0.2) | Luxembourg | 4 (0.4) |
| Belgium | 27 (2.9) | Malta | 6 (0.6) |
| Bosnia and Herzegovina | 5 (0.5) | Moldova | 3 (0.3) |
| Bulgaria | 8 (0.9) | Monaco | 1 (0.1) |
| Croatia | 26 (2.8) | Netherlands | 50 (5.4) |
| Cyprus | 4 (0.4) | Macedonia | 3 (0.3) |
| Czechia | 10 (1.1) | Norway | 8 (0.9) |
| Denmark | 11 (1.2) | Poland | 19 (2.0) |
| Estonia | 4 (0.4) | Portugal | 56 (6.0) |
| Finland | 12 (1.3) | Romania | 31 (3.3) |
| France | 24 (2.6) | Russia | 12 (1.3) |
| Georgia | 2 (0.2) | Serbia | 14 (1.5) |
| Germany | 132 (14.2) | Slovakia | 8 (0.9) |
| Greece | 45 (4.8) | Slovenia | 14 (1.5) |
| Hungary | 8 (0.9) | Spain | 73 (7.8) |
| Iceland | 1 (0.1) | Sweden | 43 (4.6) |
| Ireland | 18 (1.9) | Switzerland | 65 (7.0) |
| Israel | 6 (0.6) | Turkey | 23 (2.5) |
| Italy | 40 (4.3) | Ukraine | 7 (0.8) |
| Kazakhstan | 2 (0.2) | United Kingdom (UK) | 44 (4.7) |
| Kosovo | 3 (0.3) | Uzbekistan | 2 (0.2) |
| Latvia | 10 (1.1) | | |

**Table S2.** Questionnaire items.

| **Overall** | **n (%)** |
|---|---|
| Do you need to obtain written Informed consent for elective surgery due to legal requirements from both parents or just from one (simple procedures, low risk patients)? | |
| *Always both* | 92 (14.2) |
| *Different* | 53 (8.2) |
| *Just the one, who is present* | 502 (77.6) |
| Do you need to obtain written Informed consent for elective surgery due to legal requirements from both parents or just from one (complex procedures, higher risk patients)? | |
| *Always both parents* | 167 (25.7) |
| *Different* | 68 (10.5) |
| *Just the one, who is present* | 415 (63.8) |
| Do you know if it is legally allowed to obtain informed consent from the parent/caregiver via Internet or telephone? | |
| *Different* | 63 (9.8) |
| *No* | 432 (67.3) |
| *Yes* | 147 (22.9) |
| When ensuring a certain guideline, which may include i.e. specific appointment (time and date) for the interview,<br>secure data protected online service, asking if there is a relaxed environment without distraction, | |

| | |
|---|---|
| parents’ explicit agreement in online or telephone interview. Would this be an alternative to personal face-to-face meetings? | |
| *Different* | 52 (7.6) |
| *No* | 222 (32.3) |
| *Yes* | 414 (60.2) |
| In case of personal presence of the parent/caregiver do you routinely check the identity or legal responsibility of the parent/caregiver (i.e. ID card) | |
| *Different* | 29 (4.5) |
| *No* | 423 (65.6) |
| *Yes* | 193 (29.9) |
| Do you think it is necessary to verify the identity of the parent/guardian (i.e. ID card) | |
| *Different* | 49 (7.6) |
| *No* | 234 (36.3) |
| *Yes* | 362 (56.1) |
| What are your major concerns about online or telephone interviews? | |
| *Lack of contact* | 632 (26.8) |
| *Can't observe behavior* | 504 (21.4) |
| *No relationship* | 396 (21.4) |
| *No confidence (missing doctor-patient relationship)* | 264 (16.8) |
| *Legal requirements* | 268 (11.3) |
| *Unsure if illegal* | 291 (12.3) |
| What could be a major advantage of online/telephone interviews? | |
| *Less stressful* | 308 (23.1) |
| *Less waiting* | 470 (35.3) |
| *Standardized questionnaires* | 224 (16.8) |
| *More efficient than face-to-face* | 327 (24.6) |
